# Expression and clinical significance of angiotensin II type 1 receptor in human hepatocellular carcinoma

**DOI:** 10.3892/etm.2013.1411

**Published:** 2013-11-15

**Authors:** YUN-FEI DUAN, XIAO-DONG LI, FENG ZHU, FENG ZHANG

**Affiliations:** 1 Department of Liver Transplantation, The First Affiliated Hospital of Nanjing Medical University, Nanjing, Jiangsu 210029, P.R. China; 2 Department of Oncology, The Third Affiliated Hospital of Soochow University, Changzhou, Jiangsu 213003, P.R. China; 3 Department of Hepatobiliary Surgery, The Third Affiliated Hospital of Soochow University, Changzhou, Jiangsu 213003, P.R. China

**Keywords:** angiotensin II type 1 receptor, hepatocellular carcinoma, differentiation

## Abstract

This study aimed to investigate the expression of angiotensin II type 1 receptor (AT-1R) mRNA and the AT-1R protein in human primary hepatocellular carcinoma (PHC), and to attempt to elucidate their association with pathological and clinical characteristics. Fresh tumor and normal liver tissues were obtained from 44 patients with PHC following hepatectomies. AT-1R mRNA levels were quantitatively analyzed by quantitative polymerase chain reaction (qPCR) while the protein levels were assessed by immunohistochemistry. The expression levels of AT-1R were observed in hepatocellular carcinoma tissues and normal liver tissues. The level of AT-1R protein expression in normal liver tissues was higher compared with that in PHC tissues (P=0.0033). The AT-1R mRNA levels were higher in patients with negative hepatitis B virus surface antigen (HBsAg), normal α-fetoprotein (AFP) levels and high tumor differentiation, compared with those in patients with positive HBsAg (P=0.0005), upregulated AFP levels (P=0.0008) and poor tumor differentiation (P=0.0290). No significant correlation was identified between the expression levels of AT-1R mRNA and general characteristics such as gender, age, cirrhotic nodules, tumor size, tumor encapsulation, tumor number, carcinoma embolus, tumor metastasis or tumor recurrence. Downregulated levels of AT-1R mRNA and AT-1R protein may indicate a poor prognosis for patients with PHC.

## Introduction

Primary hepatocellular carcinoma (PHC), a prevalent cancer, is the third leading cause of cancer related mortality (1,2). Furthermore, the incidence of PHC is increasing in many countries and regions, particularly in China (2). Additionally, only ~20% of patients are eligible for curative surgery, with limited therapeutic options for those who are ineligible. Failure to achieve a timely diagnosis, in addition to the limited efficacy of palliative treatments, contributes to the poor prognosis for PHC patients. Furthermore, PHC remains a highly lethal disease due to the recurrence of metastasis, thereby leading to poor patient prognosis (3).

Due to the scarcity of efficacious testing methods, the identification of novel PHC biomarkers is necessary. To date, several studies have focused on tests that are capable of detecting and monitoring PHC, including tests for the ratio of glycosylated α-fetoprotein (AFP; L3 fraction) to total AFP, and prothrombin induced by vitamin K absence II (PIVKA II), α-fucosidase and HSP-70 levels (4). However, the specificity and sensitivity of these serological markers are low and have been demonstrated to be inadequate and impractical for the purposes of PHC screening, even when they are combined (2).

Angiotensin II (AT-II) is a major peptide hormone of the renin-angiotensin system (RAS), which is crucial for maintaining cardiovascular homeostasis and mediating diverse physiological functions such as cell growth, differentiation and apoptosis (5). The majority of AT-II actions are mediated by its two sub-receptors, which are the AT-II type 1 receptor (AT-1R) and the AT-II type 2 receptor (AT-2R) (6). These two subunits control the effects of AT-II on various organs (5,7), while AT-2R is less common than AT-1R and has been observed in fetal cells (8).

Previous studies have revealed AT-II to have major functions in several aspects of neoplastic diseases, which indicate an anti-neoplastic action for AT-II by binding to activated AT-1R (9). Activation of the AT-1R associated with tumor development may be via various pathways. AT-1R has the potential to stimulate tumor growth factors, which results in the suppression of immune function (10). AT-1R assists vascular endothelial growth factor (VEGF) to promote tumor vessel growth. Furthermore, AT-1R is capable of mediating inflammation by stimulating various inflammatory factors including interleukin 1β, tumor necrosis factor-α, plasminogen activator inhibitor-1 and adrenomedullins (11,12). These effects cause enduring tumor vessel growth, tumor invasion and metastasis, and immunosuppression, thereby leading to the development of tumors. Kawamata *et al*(13) transformed non-invasive esophageal cancer cells into AT-1R overexpressed invasive esophageal cancer cells, and suggested that nine inflammation-related genes in the cells were altered, indicating that AT-1R promoted tumor growth via inflammation-inducing factors.

Numerous studies have reported that AT-1R overexpression is potentially associated with various malignancies such as non-small cell lung cancer (14), gastric cancer (15,16), breast cancer (17,18), ovarian cancer (19), bladder cancer (20,21), pancreatic cancer (22,23) and prostate cancer (24–27). However, currently there is limited literature regarding AT-1R expression in patients with PHC and the results are frequently contradictory. Di *et al*(28) demonstrated that AT-1R was overexpressed in human hepatocellular carcinoma tissues by using immunohistochemistry, and thus concluded it was a marker reflecting the degree of malignancy of the hepatocellular carcinoma. However, Wu *et al*(29) concluded that the levels of AT-1Rs in normal tissues were markedly higher compared with those in hepatocellular carcinoma tissues by immunohistochemistry in a murine xenograft hepatocellular cancer model. Nevertheless, the two studies used traditional semi-quantitative methods, which leads to a certain degree of subjectivity and possible inaccuracy. Additionally, subgroups of PHC were not mentioned.

This study aimed to determine AT-1R mRNA and AT-1R protein levels in PHC tissues, elucidate their association with the clinicopathological characteristics of PHC and confirm the clinical value of AT-1R as a biomarker for PHC in clinical diagnosis.

## Patients and methods

### Patient enrollment and tissue samples

In total, 44 patients with PHC were enrolled between January 2007 and June 2013 in the Department of Hepatobiliary Surgery, The Third Affiliated Hospital of Soochow University (Changzhou, China). All diagnoses were verified pathologically. Clinical data were obtained by retrospective chart review. Survival was determined from the date of the initial surgery. Follow-up was available for all patients. The survival period ranged from 1–72 months (mean, 24.1±16.4 months). Of the 44 patients, 36 were male and 8 were female. The ages ranged between 28–78 years with an average age of 52 years. All enrolled patients were treated with radical surgery for PHC and received no other treatments. A section of tumor tissue 0.5×0.5×0.5 cm was obtained from each patient immediately after the surgery. Additionally a section of normal liver tissue, 0.5×0.5×0.5 cm and >5 cm away from the tumor margin was obtained. All tissue samples were fixed in 10% formalin, embedded in paraffin, and routinely stained with hematoxylin and eosin. Specimens were assessed blindly and independently by two pathologists. In case of interobserver disagreement, final decisions were achieved by general consensus. The cancer grading was determined by histology according to Edmondson’s criteria (30). Edmondson’s grade I–II was designated low-grade PHC and Edmondson grade III–IV was designated high-grade PHC. All enrolled patients provided written consent. The protocol was approved by the institutional ethics review board at Soochow University. This study complies with the principles of the Declaration of Helsinki and Good Clinical Practice Guidelines.

### Quantitative polymerase chain reaction (qPCR)

Unless indicated otherwise, all reagents for qPCR were purchased from Fermentas-China Inc. (Shenzhen, China). Total RNA was extracted from the tumor and normal tissues using an extraction reagent (Shennengbocai Inc., Shanghai, China) according to the manufacturer’s instructions. RNA samples were stored at −70°C until required. PCR was performed using a RevertAid™ First Strand cDNA Synthesis kit with PCR primers for synaptophysin designed by TaqMan^®^ Gene Expression Assays (Invitrogen, Carlsbad, CA, USA). RNA (3 μg) was reverse transcribed using the First Strand cDNA Synthesis kit with 0.5 μg oligo(dT)_16_ according to the manufacturer’s instructions. The reaction mixture was incubated at 70°C for 5 min, and subsequently at 0°C for 30 sec. The cDNA concentration was determined by spectrophotometer. Glyceraldehyde 3-phosphate dehydrogenase (GAPDH) was used as an internal control. Specific primers for AT-1R were synthesized as follows: forwards, 5′-AGACAGATGACGGCTGCTCG-3′; reverse, 5′-AACAATCTGGAACTCTCATCTCCTG-3′. Specific primers for GAPDH were synthesized as follows: forwards, 5′-GGAAGGTGAAGGTCGGAGTC-3′; reverse, 5′-CGTTCTCAGCCTTGACGGT-3′. The cycling conditions were as follows: initial denaturation at 95°C for 3 min, followed by 40 cycles at 95°C for 15 sec, and a final extension for 45 sec at 60°C. The relative level of gene expression was evaluated using 2^−ΔΔCt^.

### Immunohistochemistry

All reagents for immunohistochemistry were obtained from R&D systems, Inc. (Minneapolis, MN, USA). Tissue sections (5 μm) were deparaffinized in xylene, rehydrated in an ethanol series and subsequently treated for 30 min with 0.3% hydrogen peroxide, washed with phosphate-buffered saline (PBS) and unmasked in a citrate antigen unmasking solution for 20 min at 120°C. The sections were incubated with primary antibodies [rabbit anti-human polyclonal antibody to AT-1R (1/50)] for 1 h at room temperature. The bound primary antibodies were detected by adding secondary antibodies (peroxidase labeled goat anti-human IgG) and avidin/biotin/horseradish peroxidase complex (Dako, Carpinteria, CA, USA) for 30 min at room temperature. The sections were visualized using solid diaminobenzidine diluted with PBS, counterstained with hematoxylin and mounted. Breast cancer tissue was used as the positive control. Three independent investigators assessed the positivity of AT-1R semiquantitatively without prior knowledge of the clinical study. The intensity of cytoplasmic staining was defined as negative (stained cells, <20%) or positive (stained cells, ≥20%).

### Statistical analysis

Data were analyzed using GraphPad Prism 5 (GraphPad Software Inc., San Diego, CA, USA). The differences in AT-1R mRNA levels between PHC tissues and normal tissues were compared using the Wilcoxon test. The differences in AT-1R mRNA among various subgroups of PHC were analyzed using the non-pairing t-test. P<0.05 was considered to indicate a statistically significant difference.

## Results

### Baseline patient characteristics

Baseline patient characteristics, including gender, age, pathological grade, HBS infection status, tumor size and number as well as recurrence status are shown in Table I.

### AT-1R mRNA expression

**Figure 1:**
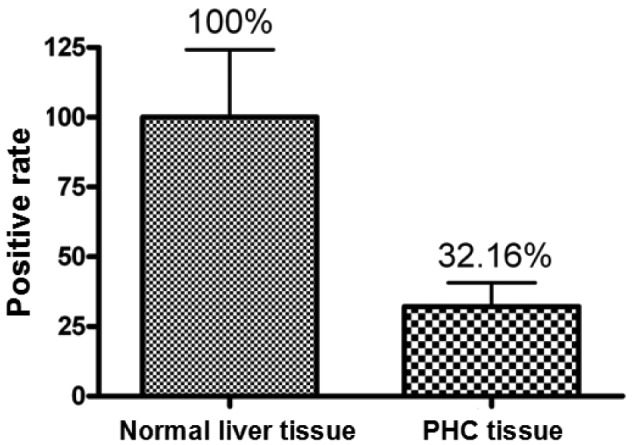
Comparison of angiotensin II type 1 receptor (AT-1R) mRNA expression in normal liver tissues and primary hepatocellular carcinoma (PHC) tissues. Relative expression rates of AT-1R mRNA expression in normal tissues and tumor tissues were 100 and 32.16%, respectively (P=0.0033).

AT-1R mRNA and GAPDH mRNA were expressed in all hepatocellular carcinoma tissues and normal liver tissues. In 40 cases (90.9%), the AT-1R mRNA expression level in normal tissues was higher compared with that in the tumor tissues, and the opposite was observed for the other four cases. The difference was considered to be statistically significant (P=0.0033). The relative expression rates of AT-1R mRNA in normal tissues and in tumor tissues were 100 and 32.16%, respectively (Fig. 1).

### AT-1R protein expression

**Figure 2:**
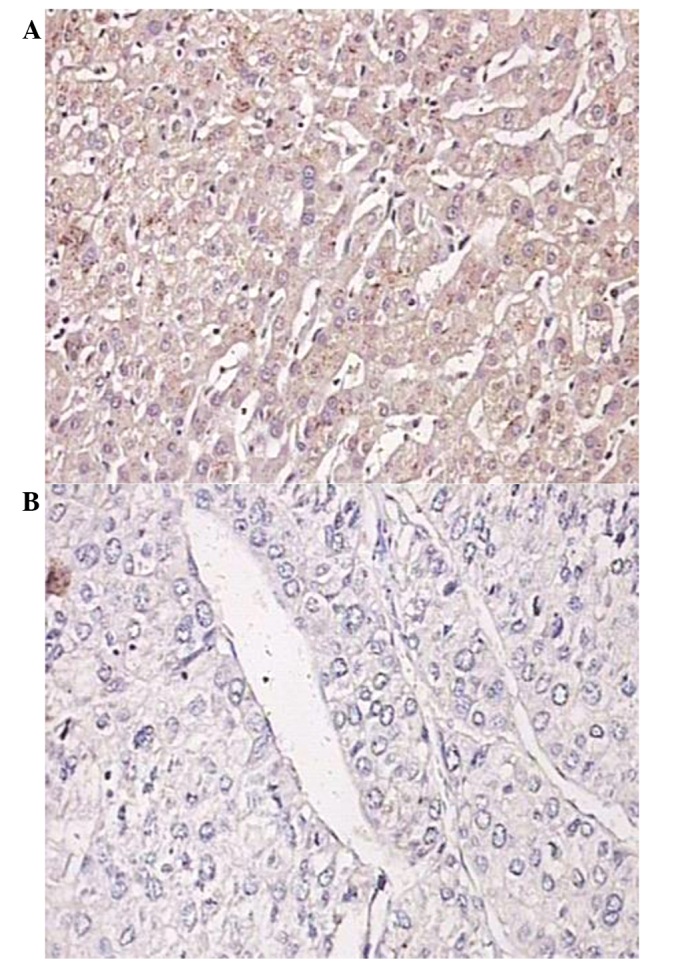
Expression of angiotensin II type 1 receptor (AT-1R) protein (magnification, ×200). (A) AT-1R is mainly expressed in the cytoplasm presenting as brown granules in normal tissue. (B) AT-1R granules are observed in PHC tissue.

The majority of the AT-1R expression was detected in the cytoplasm. The number of positively stained cells and the staining density were markedly higher in the normal tissues compared with those in the tumor tissues (Fig. 2).

### Correlation between AT-1R mRNA expression and clinicopathological factors

The AT-1R mRNA expression levels in cases which were positive for HBsAg infection (40/44) were markedly higher compared with those in cases which were negative for HBsAg infection (4/44) (P=0.0005). The AT-1R mRNA expression levels in cases with normal AFP levels (6/44) were markedly higher compared with those in cases with aberrantly increased AFP levels (38/44) (P=0.0008). The AT-1R mRNA expression levels in cases with Edmondson’s pathological grade I–II (24/44) were markedly higher compared with that in cases with Edmondson’s pathological grade III–IV (20/44) (P=0.0290; Table II).

**Figure 3:**
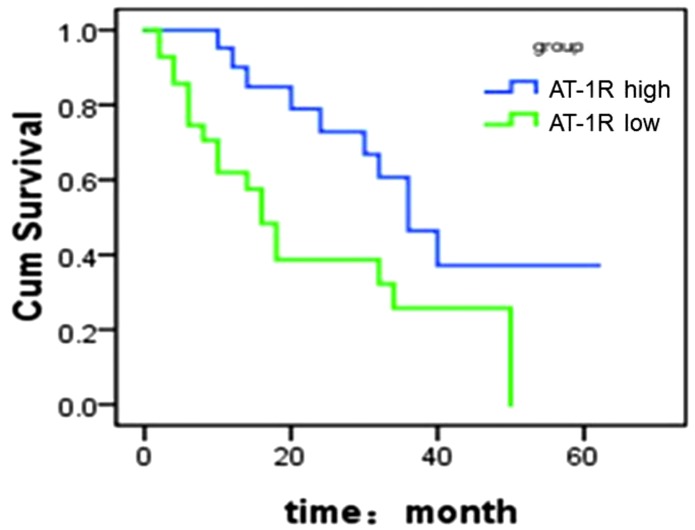
Comparison of overall survival rate of patients with high and low expression of angiotensin II type 1 receptor (AT-1R) mRNA in hepatocellular carcinoma specimens. Cum, cumulative.

Notably, the data from the 5-year follow-up demonstrated a correlation between AT-1R mRNA expression level and patient survival rate. In cases with high levels of AT-1R mRNA expression, the 3-year survival rate was 46%, with a median survival time of 35.5 months. However, in cases with low levels of AT-1R mRNA expression, the 3-year survival rate was 26%, with a median survival time of 15.6 months (Fig. 3).

However, no correlation was observed between AT-1R mRNA expression and other clinical features, such as age, gender, tumor number, tumor size, cirrhosis status, tumor encapsulation, cancerous embolus and recurrence (Table II).

## Discussion

PHC represents a paradigm of the correlation between the tumor microenvironment and tumor development (31). It has been demonstrated that controlling the growth of tumor vessels is an important modality for the treatment of PHC.

To date, the function of the VEGF family for generating tumor vessel growth has been relatively well clarified (32). VEGF is crucial in the development of PHC by inducing tumor vessel growth in the early stages, and VEGF levels have been observed to correlate positively with microvessel density (33). However, the mechanism by which VEGF regulates PHC cells growth has not been fully elucidated. Fujiyama *et al*(34) demonstrated that AT-II promoted the expression of VEGF by endothelial cells.

The results of the present study indicated that the level of AT-1R expression in normal liver tissues was higher than that in tumor tissues, potentially due to that fact that the majority of PHC cases had HBV-related hepatocirrhosis (36/44). This indicates that the upregulation of AT-1R expression is correlated with hepatocyte proliferation. Furthermore, it was observed that the AT-1R mRNA expression level correlated negatively with hepatocyte differentiation. Once PHC formed an invasive cancer, which broke through the basement membrane, AT-1R expression was downregulated. Takeda *et al*(35) demonstrated that positive rates of AT-1R expression in well-differentiated, moderately differentiated and poorly differentiated squamous cell carcinomas were 81, 72 and 0%, respectively, which were consistent with the results of the present study.

De Paepe *et al*(36) applied immunohistochemistry and *in situ* hybridization to investigate the expression of AT-1R in various stages of breast cancer, and the results revealed that AT-1R was overexpressed in neoplasms with a relatively low level of malignancy. These results are consistent with those of the present study. De Paepe *et al*(36) hypothesized that AT-1R was an important mediator for the precursors of breast cancer but not a necessary protein for invasive breast cancer. Similarly, we considered that AT-1R is unnecessary for PHC. Lower expression levels of AT-1R in PHC tissues leave the blood supply for PHC cells unaffected by AT-II, leading to the sustained growth of PHC; this is a difference between PHC and normal vessels.

Notably, the data from the present study demonstrated that patients with higher levels of AT-1R mRNA expression have an improved survival rate, indicating that AT-1R is a novel prognostic factor in hepatic carcinoma.

In conclusion, AT-1R mRNA is expressed in normal liver tissue and PHC tissue. AT-1R mRNA levels correlate negatively with the degree of malignancy of PHC, which is a potential cause of the increased blood supply in PHC tissues. AT-1R mRNA expression correlates with PHC cell differentiation, but does not correlate with gender, age, hepatocirrhotic nodules, tumor size, tumor number, cancerous embolus, tumor encapsulation or tumor recurrence. These results suggest that AT-1R expression correlates with PHC development, and inhibits AT-1R expression prior to invasive tumor formation, which may prevent PHC from growing progressively. Future studies concerning the correlation between AT-1R and other ligands are warranted.

## Figures and Tables

**Table I:** Baseline patient characteristics (n=44).

Clinicopathologic factors	No. patients	%
Gender		
Female	36	81.8
Male	8	18.2
Age (years)		
Average	52	
Range	28–78	
Edmondson’s pathological grade		
I–II	24	54.5
III–IV	20	45.5
HBsAg infection		
Positive	40	90.9
Negative	4	9.1
AFP (ng/ml)		
≤20	6	13.6
>20	38	86.4
Hepatocirrhotic nodule (cm)		
≤3	34	77.3
>3	10	22.7
Tumor size (cm)		
≤5	8	18.2
>5	36	81.9
Tumor encapsulation		
Yes	26	59.1
No	18	40.9
Tumor number		
Single	34	77.3
Multiple	10	22.7
Cancerous embolus		
Yes	14	31.8
No	30	68.2
Recurrence		
Yes	10	22.7
No	34	77.3

HBsAg, hepatitis B virus surface antigen; AFP, α-fetoprotein.

**Table II:** Correlation between the clinicopathological factors of PHC patients and AT-1R levels in tumor tissues (n=44).

Clinicopathological factor	Cases	AT-1R/GAPDH (Mean ± SD)	t-value	P-value
Gender				
Male	36	0.3199±0.4104	0.0407	0.9680
Female	8	0.3292±0.4140		
Age (years)				
≤50	18	0.3341±0.2943	0.1183	0.9070
>50	26	0.3130±0.4728		
HBsAg				
Negative	4	1.1660±0.8565	4.1670	0.0005^a^
Positive	40	0.2371±0.2378		
AFP (ng/ml)				
≤20	6	0.9730±0.7784	3.9340	0.0008^a^
>20	38	0.2188±0.1961		
Hepatocirrhotic nodule (cm)				
≤3	34	0.3426±0.4142	0.4431	0.6624
>3	10	0.2504±0.3873		
Tumor size (cm)				
≤5	8	0.1225±0.0714	1.1030	0.2829
>5	36	0.3659±0.4317		
Tumor encapsulation				
No	18	0.1999±0.1491	1.1960	0.2456
Yes	26	0.4059±0.4979		
Edmondson’s pathological grade				
I–II	24	0.4881±0.4841	2.3520	0.0290^a^
III–IV	20	0.1218±0.0867		
Tumor number				
Single	34	0.3323±0.4257	0.2250	0.8243
Multiple	10	0.2853±0.3429		
Cancerous embolus				
No	30	0.3840±0.4636	1.0730	0.2962
Yes	14	0.1878±0.1759		
Recurrence				
No	34	0.3413±0.4370	0.4163	0.6816
Yes	10	0.2546±0.2710		

a P<0.05.

PHC, primary hepatocellular carcinoma; AT-1R, angiotensin II type 1 receptor; GAPDH, glyceraldehyde 3-phosphate dehydrogenase; HBsAg, hepatitis B virus surface antigen; AFP, α-fetoprotein.
